# Atomic-Scale Description of the Magnetic TiN|FeCo Multilayers

**DOI:** 10.1021/acsomega.4c01288

**Published:** 2024-05-28

**Authors:** Rodrigo Ponce-Pérez, Carlos Antonio Corona-García, Jose Mario Galicia Hernandez, Armando Reyes-Serrato, Joseph Perry Corbett, Jonathan Guerrero-Sánchez

**Affiliations:** † Centro de Nanociencias y Nanotecnología, Universidad Nacional Autónoma de México, Ensenada, Baja California 22860, México; ‡ Department of Physics, College of Arts and Science, Miami University, 501 E High St, Oxford, Ohio 45056, United States

## Abstract

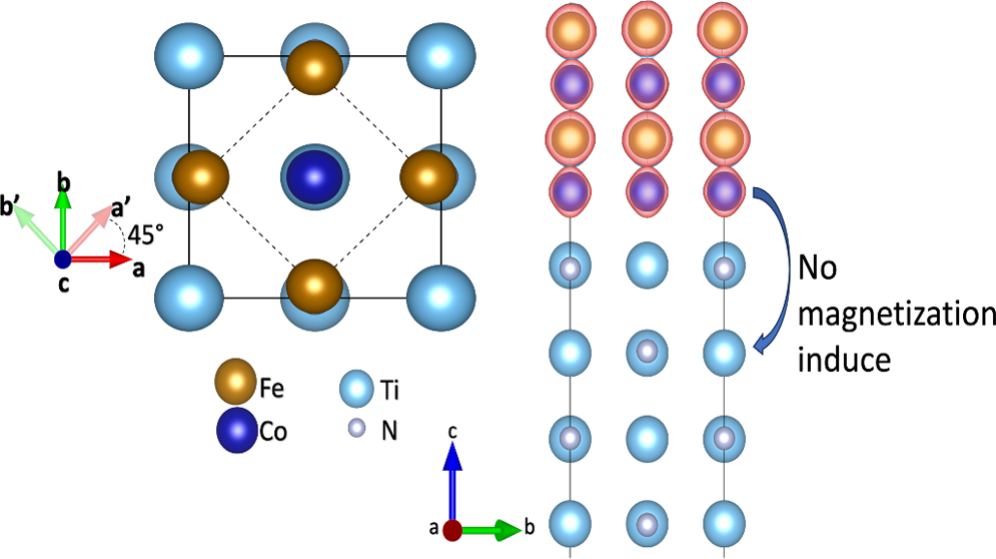

Motivated by the experimental findings of Wolff et al., we investigated the TiN|FeCo multilayers at the atomic scale. Four different models were employed to investigate the interface, considering both Fe and Co surface terminations of the FeCo compounds. The interface formation energy formalism was employed to study the thermodynamic stability of these models. The results show that an interface mediated by Co atoms is most likely to appear in the experiment. Also, the Fe surface termination is more viable than a Co surface termination. The magnetic moments of Co at the interface are 1.48 μ_B_/atom, which denotes a decay compared to bulk (1.76 μ_B_/atom). Besides, Ti acquires a very small induced magnetization of −0.05 μ_B_/atom. Our proposed atomistic model of the TiN|FeCo multilayer system fits perfectly with the structure obtained in experiments, and it is a step forward in the theoretical-experimental design of wear-resistant coatings with outstanding magnetic and mechanical properties.

## 1 Introduction

Titanium nitride (TiN) is a compound that crystallizes in the face-centered cubic (FCC) structure without magnetic characteristics; it belongs to the *Fm*3̅*m* space group.^1,2^ It is well-known as a sound engineering material, with a high electron conductivity and mobility, low work function, and high melting point.^3,4^ TiN possesses a high hardness (2000 kg/mm^2^) and a high decomposition temperature (2949 °C).^5^ TiN has been widely used as a wear-resistant coating because of its high-temperature stability, chemical stability, and mechanical properties.^6−8^ TiN also exhibits good biocompatibility and tribological properties, which, together with its mechanical properties, makes it an excellent option for medical applications like orthopedic prostheses and medical implants.^5^ More recently, in the field of plasmonics.^9−11^ Some other critical applications include the design of diffusion barriers in microelectronic devices,^12−14^ decorative coatings,^15,16^ and ohmic contacts,^17,18^ among others. This material is also used for wearing metal machine components, which is crucial for controlling the life of machine components^19,20^ to avoid any loss of dimensions and functionality.

On the other hand, the FeCo alloy is a highly magnetic material and crystallizes in the cubic structure with the *Pm*3̅m space group.^21^ It is considered a soft magnetic material that operates at high temperatures. These kinds of materials can be easily magnetized and demagnetized by the influence of a small external field. In general, the material has a combination of outstanding properties such as high saturation magnetization, high Curie temperature (>1500 K),^22^ low magnetocrystalline anisotropy, and good strength.^23,24^ The reported values for the monocrystalline anisotropy and saturation magnetization are equal to 107 and ∼1581 emu/cm^3^, respectively,^25^ which are around 50% higher than the ones observed in FePt alloys. These two properties are essential for developing diverse high-density recording media.^25^ Its high saturation magnetization makes FeCo a good candidate for coating carbon nanotubes (CNTs) and for further use in biomedical applications.^26^ On the other hand, FeCo reduces the excessive conductivity in two-dimensional (2D) MXenes. In this way, using FeCo can reduce the ultrahigh conductivity in MXenes, enhancing the magnetic loss mechanism and bringing better impedance-matching conditions.^27^ These outstanding properties will allow the design of high-performance microwave absorption and electromagnetic interference shielding materials.^27−29^

Finally, it is possible to combine TiN and FeCo in the form of multilayers to build wear-resistant coatings that can simultaneously supply outstanding mechanical properties (TiN) as well as ferromagnetic properties (FeCo).^30,31^ These can be tuned by controlling the layers’ thickness.^31^ In this way, these multilayers are suitable for applications in coatings for remote-interrogable wear sensors based on the inverse magnetostrictive effect^30^ and some other sensors based on magnetic properties, such as noncontact wear^31^ and magnetoelectric sensors^31^ and in biomagnetic imaging^31^ since this heterostructure combines the functionalities described above.

In this work, we have performed a detailed atomic-scale analysis of the TiN|FeCo multilayers to determine the exact atomic configuration of the multilayer system described in the experimental work of Wolff et al.^31^ We focus on determining the most stable interfaces based on the interface formation energy calculations. This paper is organized as follows: Section 2 describes the computational methods used for performing our calculations and briefly describes the interface formation energy formalism. The results are presented and discussed in Section 3, and finally, in Section 4, we make conclusions.

## 2 Computational Methods

The multilayer TiN|FeCo along the [001] direction is investigated by spin-polarized total-energy calculations. The calculations were performed within the periodic density functional theory (DFT) framework as implemented in the Vienna Ab Initio Simulation Package (VASP) code.^32−35^ The exchange-correlation energy was treated within the generalized gradient approximation (GGA) with Perdew–Burke–Ernzerhof parametrization.^36^ Although some atoms of our systems have d-orbitals, the structural and magnetic properties of TiN and FeCo are well described using standard DFT;^37−42^ therefore, the use of the Hubbard correction (DFT + *U*) is not considered in this work.

The electron–ion interactions were modeled by the Projector-Augmented Wave (PAW) pseudopotentials^43,44^ with 440 eV as the energy cutoff. Convergence is achieved when the energy differences are less than 1 × 10^–4^ eV. Also, in the geometry optimization, the force components must be smaller than 0.01 eV/Å. The supercell method is employed to simulate the interface. Each supercell is formed by a slab and a vacuum space larger than 20 Å to avoid interactions between periodic slabs. Each slab is formed by a central TiN layer (11 monolayers), where the FeCo slab (10 monolayers) is placed on top and bottom of the TiN layer in a 1 × 1 periodicity. Since inversion is included, we have two equivalent surfaces in each supercell. A *k*-point grid of 11 × 11 × 1 was used for sampling the reciprocal space by following the Monkhorst–Pack scheme.^45^

### 2.1 Interface Formation Energy Formalism

Since we are interested in investigating the most stable interfaces in the TiN|FeCo multilayer system, we have to employ the interface formation energy (IFE) formalism, which is independent of the number of atoms and depends only on the chemical potential of the constituent species. The IFE formalism can be adapted to our system following the ref (46) as follows
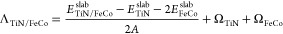
1where *E*_TiN/FeCo_^slab^ is the total energy of the TiN|FeCo interface, and *E*_TiN_^slab^ and *E*_FeCo_^slab^ are the total energies of the TiN and FeCo isolated slabs, respectively. *A* is the area of the supercell, and Ω_TiN(FeCo)_ is the surface formation energy (SFE) for the TiN (FeCo) slabs, defined as
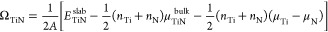
2
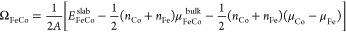
3with *n*_*i*_ and μ_*i*_ as the number of atoms and chemical potential of the *i*th species.

With this, we plot the IFE in a three-dimensional (3D) graph by varying the μ_Ti_ from Ti-rich conditions (μ_Ti_ = μ_Ti_^bulk^) to Ti-poor conditions (μ_Ti_ = μ_Ti_^bulk^ – Δ*H*_f_^TiN^) and varying the μ_Co_ from Co-rich conditions (μ_Co_ = μ_Co_^bulk^) to Co-poor conditions (μ_Co_ = μ_Co_^bulk^ – Δ*H*_f_^FeCo^). With Δ*H*_f_, the formation enthalpy, the calculated Δ*H*_f_^TiN^ and Δ*H*_f_^FeCo^ are 3.89 and 0.11 eV, respectively.

## 3 Results and Discussion

### 3.1 Structural Properties

**Figure 1:**
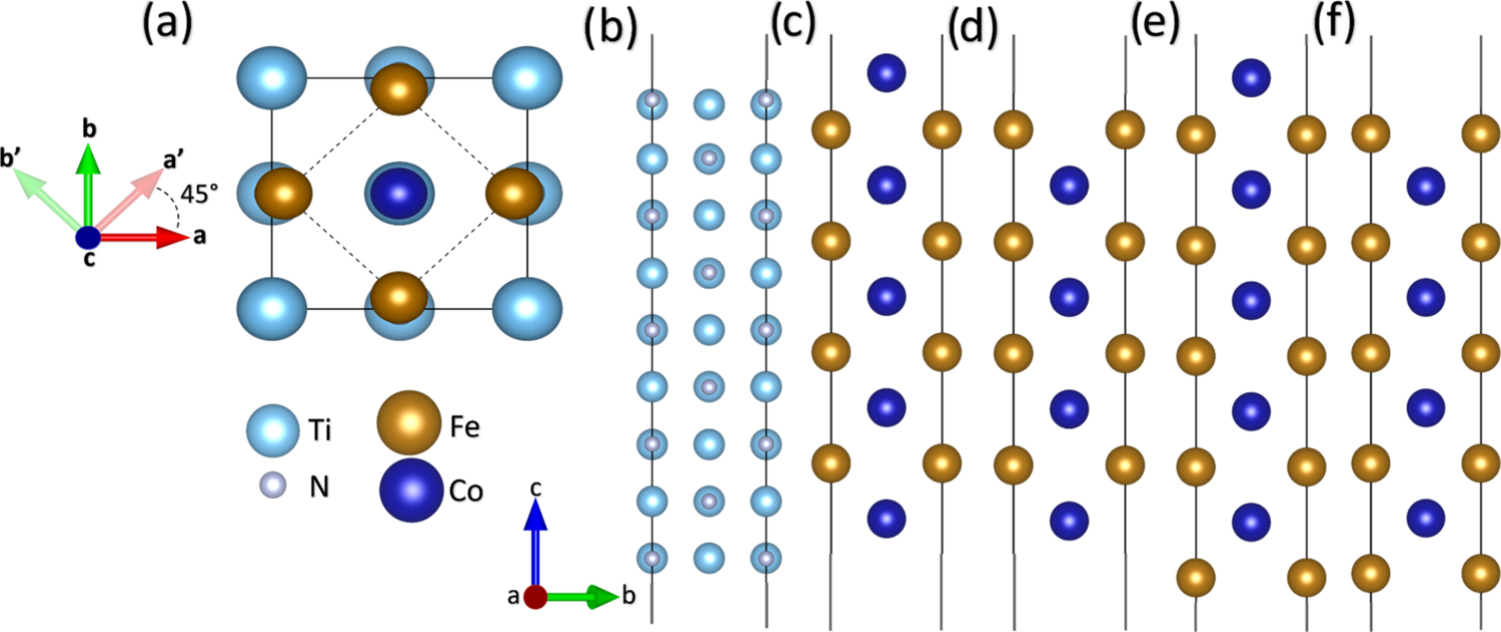
Surface models of TiN and FeCo surfaces. (a) Top view of the TiN and FeCo surfaces, showing the 45° rotation needed to shorten the mismatch parameters of both structures, (b) TiN-terminated surface, and (c–f) possible atomic configurations of FeCo surfaces. (c) Co|Co, (d) Co|Fe, (e) Fe|Co, and (f) Fe|Fe.

For multilayers of TiN and FeCo, the FeCo layers grow pseudomorphically on a TiN lattice in limited FeCo thicknesses <1 nm.^31,47,48^ FeCo’s magnetic properties are known to depend on layer thickness and, in the pseudomorphic limit, become particularly interesting.^31,48,49^ To model a superlattice of TiN and ultrathin FeCo, Wolff et al.^31^ utilized a uniformly strained pseudomorphic cubic FeCo model atop relaxed TiN, which they used to simulate their experimental diffraction data resulting in good agreement of X-ray and electron diffraction data.^31^ Despite observations of lattice variation (up to ∼1%) of the FeCo under semiperiodic tensile and compressive strains, which accompany pseudomorphic films, the uniform cubic model matched the diffraction positions remarkably well, where the lattice variation only broadened the diffraction spots. Our work follows suit, utilizing a cubic FeCo model interfaced with TiN. The calculated lattice parameter for the TiN and FeCo compounds resulted in *a*_TiN_= 4.25 Å^37,38^ and *a*_FeCo_ = 2.84 Å,^39,50^ in agreement with previous experimental reports, as shown in Table 1. To construct the pseudomorphic interface with a minimal lattice mismatch, the FeCo alloy rotates 45° concerning the TiN, as shown by Wolff et al.,^31^ see Figure 1a. With the following crystallographic relationships, we use [100]_FeCo_||[110]_TiN_ and [001]_FeCo_||[001]_TiN_. With this rotation, the theoretical mismatch between lattice parameters is approximately 5.6%, with an observed value of 5.2%.^31^ The mismatch is not big enough to induce phase transitions, distortions, or strain-induced defects in the TiN|FeCo system. Also, Klever et al. have experimentally demonstrated epitaxial growth between TiN and FeCo.^31,47,48^

**Table 1:** Lattice Constants and Bond Lengths for the TiN and FeCo Bulk Structures^a^

surface	lattice constant (Å)	Ti–N bond length (Å)	Fe–Co bond length (Å)
TiN	*a* = 4.25	2.12	
FeCo	*a* = 2.83		2.45
other works			
(a) TiN: *a* = 4.24 (exp.),^51^*a* = 4.24 (theory).^37,38^			
(b) FeCo: *a* = 2.85 (exp.),^52^*a* = 2.84, 2.85 (theory).^39,50^			

a Data from previous reports (values in parentheses) are added for comparison purposes.

The crystal structure of the TiN compound reveals that just one termination is possible for the (001) surface, which is formed by the combination of Ti and N atoms in the same monolayer (Figure 1b). On the other hand, the crystal structure of FeCo evidence four different terminations: the Co|Co surface (Figure 1c), where the lower and upper layers are composed of Co atoms; the Co|Fe surface (Figure 1d), where the lower and upper layers are composed of Co and Fe atoms, respectively; the Fe|Co surface (Figure 1e), where upper and lower surface terminations are composed of Fe and Co atoms, respectively; and the Fe|Fe surface (Figure 1f), where both lower and upper terminations are composed of Fe atoms.

**Figure 2:**
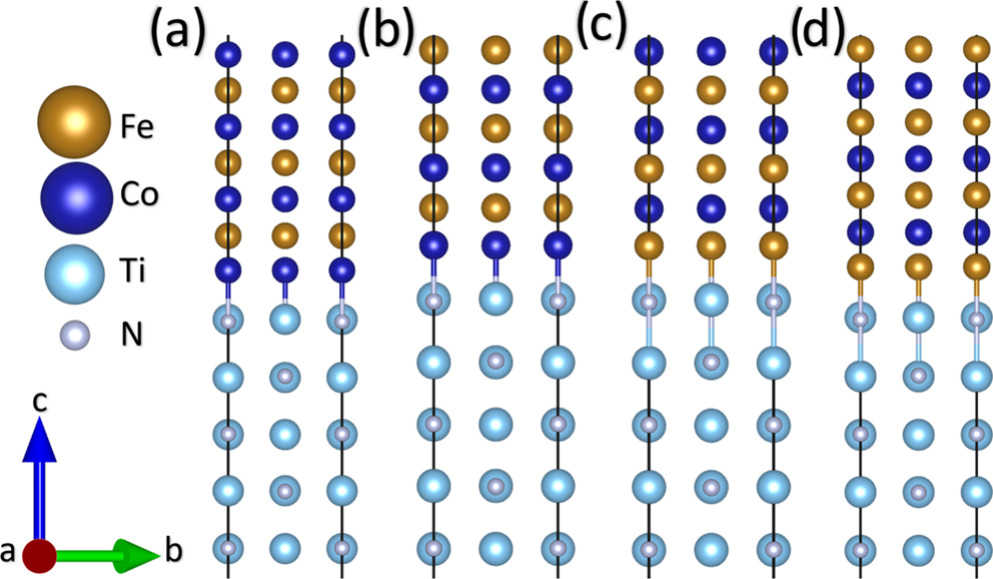
Side views of the four different TiN-FeCo possible interfaces: (a) TiN-Co|Co, (b) TiN-Co|Fe, (c) TiN-Fe|Co, and (d) TiN-Fe|Fe.

Once we have considered all of the possible surface terminations of both TiN and FeCo(001) surfaces, we now construct the interfaces. According to the experimental results, the multilayered system is formed by alternated slabs of TiN and FeCo along the [001] direction. We focus on the interface region by considering both compounds’ possible surface terminations. From this, four different structures with different interfaces can be formed. These structures are depicted in Figure 2.

Figure 2 shows the four relaxed atomistic models corresponding to the possible interfaces. The system with the interface formed by TiN–Co and terminated with Co atoms, namely TiN–Co|Co, is shown in Figure 2a. Figure 2b shows the atomistic model of the TiN–Co|Fe interface, with the interface formed by TiN–Co and terminated with Fe atoms. In both structures, the bond distance between N and Co species at the interface is 1.91 Å, and the distance between the Co and the nearest Ti atoms is 2.80 Å. On the other hand, the TiN–Fe|Co system is shown in Figure 2c with TiN–Fe atoms at the interface and terminated with Co atoms. Finally, Figure 2d depicts the TiN–Fe|Fe structure with TiN–Fe atoms at the interface and terminated with Fe atoms. In these last two structures, the bond length between Fe and N species is 1.92 Å, and the distance between the Fe and the nearest Ti atoms is 2.81 Å. There is no direct evidence of recrystallization or interdiffusion due to temperature effects, as experimentally reported by Wolff et al.^31^ The structural stabilization of the different interfaces is attributed to the bond length between Co–N and Fe–N species, which leads to the possible formation of cobalt nitride or iron nitride species at the interface of the multilayers, as previously suggested.^40,41,53,54^

### 3.2 Thermodynamic Stability

**Figure 3:**
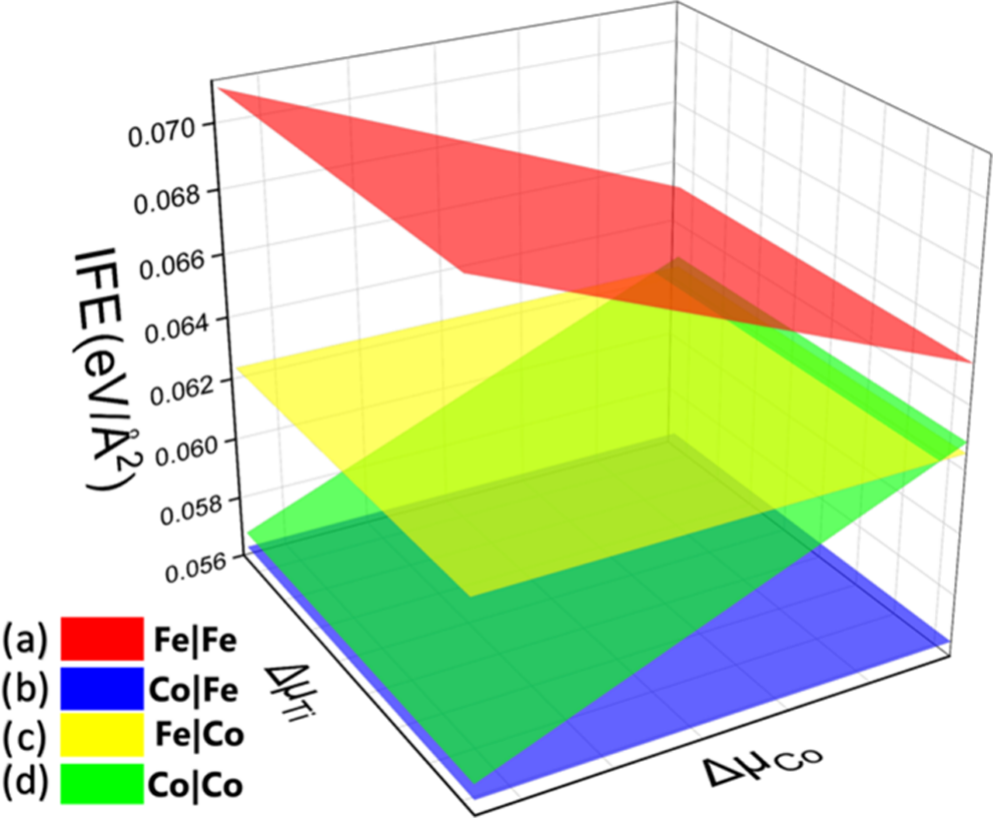
3D IFE plot of four possible interfaces in the TiN|FeCo multilayers. Red plane (a) corresponds to the TiN-Fe|Fe, the blue plane (b) belongs to the TiN-Co|Fe, the yellow plane (c) is for the TiN-Fe|Co, and the green plane (d) represents the TiN-Co|Co interfaces.

Once considering the different possible interfaces for the TiN/FeCo system, we employed the IFE formalism to find the most thermodynamically stable structure over a certain range of chemical potentials. Figure 3 shows the IFE plot for the four interfaces. Each plane represents a different interface. The most stable interfaces are the ones with the lowest IFE. The chemical potential varies from Ti-rich (right part in the Δμ_Ti_ axis) to Ti-poor (left part in the Δμ_Ti_ axis) conditions and Co-rich (left part in the Δμ_Co_ axis) to Co-poor (right part in the Δμ_Co_ axis) conditions. Figure 3 shows that the less stable (higher IFE values) multilayer interface is TiN–Fe|Fe, and the second less stable structure is TiN–Fe|Co for almost the entire growth range. Notice that these structures share the same interface with Fe atoms but have different surface terminations. Therefore, the interface mediated by Fe atoms results to be unstable.

On the other hand, the most stable structure is the one that corresponds to the TiN–Co|Fe multilayer system over the whole range of chemical potentials. Notice that at Co-poor conditions and the entire range of μ_Ti_, the TiN–Co|Co model has an energy difference of 0.4 meV compared to the TiN–Co|Fe multilayer system. This fact indicates double degeneracy in the IFE. Therefore, both structures can appear. Notice that both models have the same interface with Co but possess different surface terminations. The most stable is the Fe-terminated one.

### 3.3 Electronic and Magnetic Properties

**Figure 4:**
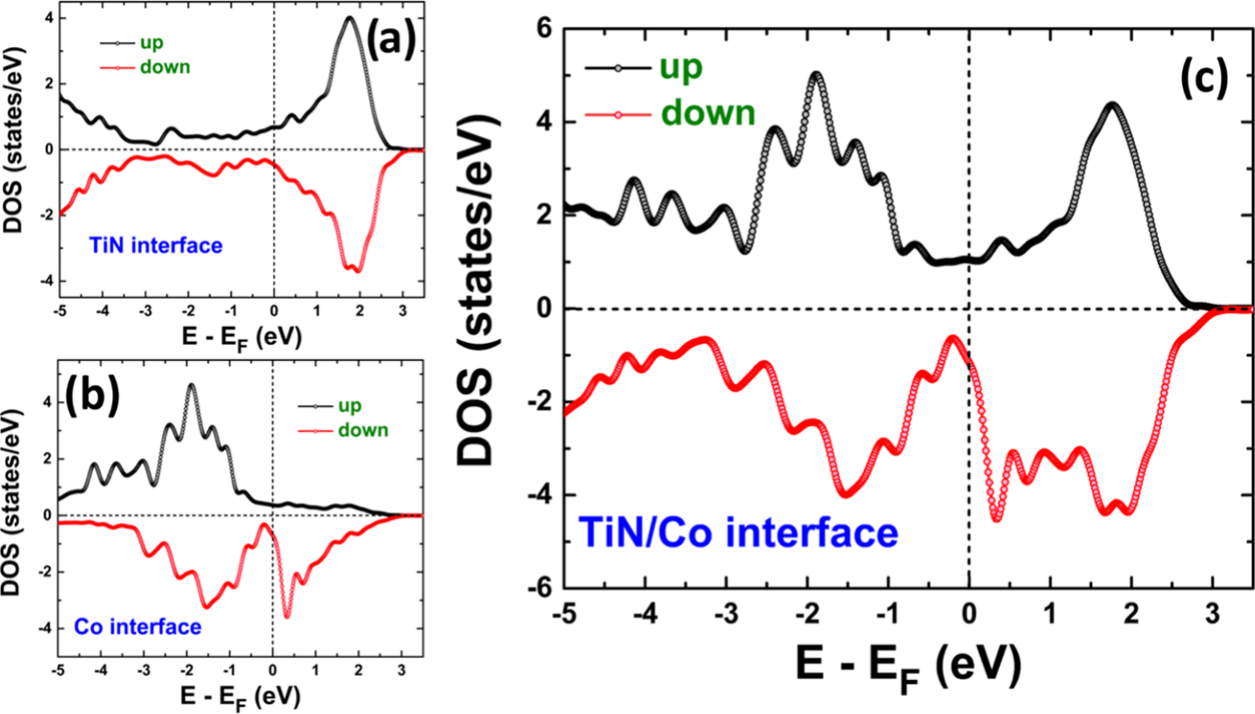
Partial density of states of the atoms at the interface. (a) Ti and N atoms, (b) Co atoms, and (c) Ti, N, and Co atoms.

We investigated the electronic properties of the most stable interface by calculating the density of states (DOS), as shown in Figure 4. In all cases, the energy reference is set to the Fermi level, with positive (negative) values along the DOS axis corresponding to spin up (down). Figure 4a,b shows the contribution of the interface TiN and Co layers to the DOS (see Figure 2b), respectively. A metallic character is observed in TiN (Figure 4a). Also, spin-up and -down channels are symmetric, which denotes their nonmagnetic nature. However, the slight asymmetries are associated with Ti atoms acquiring a tiny induced magnetic moment of −0.05 μ_B_. About the Co layer (Figure 4b), the DOS shows metallic behavior with asymmetric spin channels denoting their ferromagnetic nature. It is worth mentioning that at the interface, Co experiences a reduction in magnetic moment (1.48 μ_B_) compared to the FeCo bulk structure (1.76 μ_B_). Figure 4c depicts the DOS for the total interface formed by TiN and Co layers, where a metallic and a ferromagnetic nature is evident.

**Figure 5:**
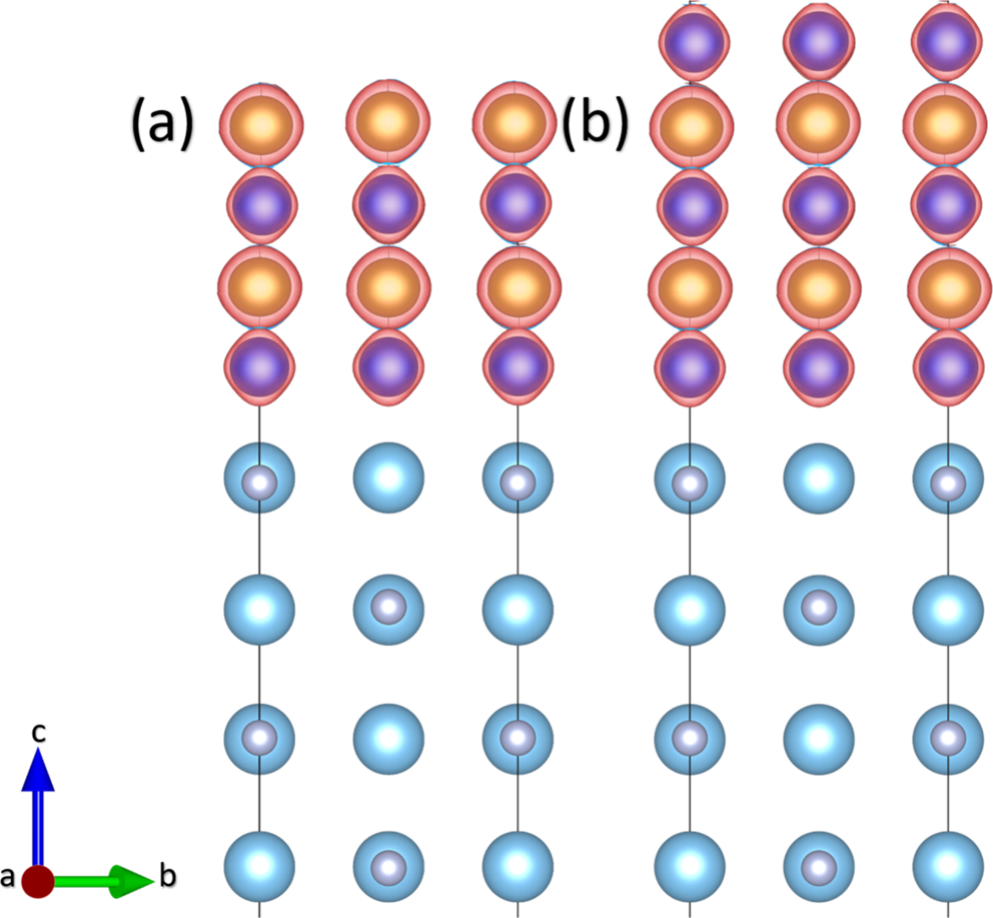
Spin density isosurfaces for (a) Co|Fe-TiN-Co|Fe and (b) Co|Co-TiN-Co|Co interfaces. For the sake of visualization, we show only four layers of TiN, four layers of FeCo for the Co|Fe structure, and five layers of FeCo for the Co|Co structure. Gray, light blue, blue, and orange spheres represent N, Ti, Co, and Fe atoms.

We also calculated the spin density of the most stable interface considering both Co- and Fe-terminated FeCo surfaces, as shown in Figure 5. The total magnetic moment ranges from 89 to 100 μ_B_ in the four proposed structures. The magnetic character in the junction comes from the Fe (2.45–2.89 μ_B_) and Co (1.72–1.84 μ_B_) atoms. Although the Ti-layer interface gets magnetized by proximity, the TiN layers remain metallic and nonmagnetic, allowing access to interlayers with bifunctionality (magnetism and strong mechanical properties). A comparison of our data to previous experimental measurements is difficult. While a similar multilayer stack was produced,^49^ the magnetic measurements were normalized to the film’s total thickness, removing the possibility of estimating the μ_*B*_ per atomic species.

**Figure 6:**
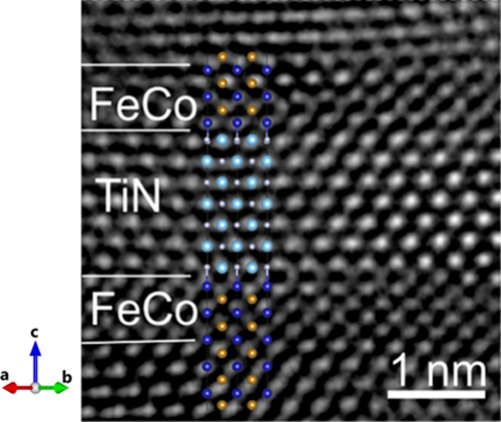
Comparison of the two most stable atomistic models and HRTEM micrograph of the Co|Fe–TiN–Co|Fe multilayers with Co atoms at the interface and Fe termination. The HRTEM micrograph is reprinted from ref (31), Copyright (2021), with permission from Elsevier.

A comparison of our DFT simulations to atomically resolved imaging enables strong validation for our theoretical model to predict the spin–split density of states alongside magnetic moments per atomic species. Figure 6 presents an atomically resolved cross-sectional transmission electron microscopy (TEM) image. Overlaid in the TEM image of Figure 6 are the atomistic models of the most stable Co|Fe–TiN–Co|Fe multilayers. The overlay shows that the atomistic model agrees well with the experiment multilayer. Moreover, experimentally from C_s_-corrected atomically resolved imaging compared with simulated TEM imaging demonstrated an intensity of variation between Fe and Co. This subtle variation of Fe and Co intensity was resolved, demonstrating Co–TiN interfacing in excellent agreement with our lowest energy structures spanning the chemical potential variation. The minor discrepancies in the theoretical overlay of experimental TEM imaging on multilayers can be attributed to the poor long-range crystallinity resulting in the smearing of atomic positions. The superlattice resulted in highly c-textured layers of FeCo and TiN consisting of at least three rotational domains. Alternatively, atomic position variation due to the tensile and compressive strains at the interfaces within the superlattice could also create minor discrepancies.^31^

## 4 Conclusions

An atomic-scale description of the structure and the electronic and magnetic properties of the TiN|FeCo multilayers was carried out by using ab initio calculations. The structural analysis of four possible interfaces between the TiN and FeCo surfaces shows that the most stable interface, for almost the whole growth range, is when TiN forms bonds with Co atoms, and the FeCo surface is Fe-terminated. On the other hand, in the range corresponding to Ti-rich and Co-poor conditions, we observed a double degeneracy in energies between the TiN–Co|Fe and TiN–Co|Co heterostructures, with an energy difference of 0.4 meV. The proposed system fits perfectly with the experimental high resolution TEM (HRTEM) micrograph, with a small mismatch in atomic positions. Still, this discrepancy is probably due to the short-range crystallization of the FeCo layers. Our spin density analysis revealed that although FeCo is a strong ferromagnetic material, it just induces magnetism to the first Ti layer in TiN, and the remaining layers are metallic and nonmagnetic. The results here evidence bifunctionality in these multilayers, where it is possible to take advantage of the intrinsic magnetism of FeCo and the outstanding mechanical properties of TiN for their applications in wear-resistant coatings and magnetoelectric sensors.

## Data Availability

The data supporting this study are available in the published article.
